# Posttraumatic stress, anxiety and depression symptoms in patients during the first year post intensive care unit discharge

**DOI:** 10.1186/cc8870

**Published:** 2010-02-08

**Authors:** Hilde Myhren, Øivind Ekeberg, Kirsti Tøien, Susanne Karlsson, Olav Stokland

**Affiliations:** 1 Intensive Care Unit, Ulleval, Oslo University Hospital, Kirkeveien 177, 0407 Oslo, Norway; 2 Department of Acute Medicine, Ulleval, Oslo University Hospital, Kirkeveien 177, 0407 Oslo, Norway; 3 Department of Behavioural Sciences in Medicine, Institute of Basic Medical Sciences, Faculty of Medicine, University of Oslo, Sognsvannsveien 9, 0373 Oslo, Norway

## Abstract

**Introduction:**

To study the level and predictors of posttraumatic stress, anxiety and depression symptoms in medical, surgical and trauma patients during the first year post intensive care unit (ICU) discharge.

**Methods:**

Of 255 patients included, 194 participated at 12 months. Patients completed the Impact of Event Scale (IES), Hospital Anxiety and Depression Scale (HADS), Life Orientation Test (LOT) at 4 to 6 weeks, 3 and 12 months and ICU memory tool at the first assessment (baseline). Case level for posttraumatic stress symptoms with high probability of a posttraumatic stress disorder (PTSD) was ≥ 35. Case level of HADS-Anxiety or Depression was ≥ 11. Memory of pain during ICU stay was measured at baseline on a five-point Likert-scale (0-low to 4-high). Patient demographics and clinical variables were controlled for in logistic regression analyses.

**Results:**

Mean IES score one year after ICU treatment was 22.5 (95%CI 20.0 to 25.1) and 27% (48/180) were above case level, IES ≥ 35. No significant differences in the IES mean scores across the three time points were found (*P *= 0.388). In a subgroup, 27/170 (16%), patients IES score increased from 11 to 32, *P *< 0.001. No differences in posttraumatic stress, anxiety or depression between medical, surgical and trauma patients were found. High educational level (OR 0.4, 95%CI 0.2 to 1.0), personality trait (optimism) OR 0.9, 95%CI 0.8 to 1.0), factual recall (OR 6.6, 95%CI 1.4 to 31.0) and memory of pain (OR 1.5, 95%CI 1.1 to 2.0) were independent predictors of posttraumatic stress symptoms at one year. Optimism was a strong predictor for less anxiety (OR 0.8, 0.8 to 0.9) and depression symptoms (OR 0.8, 0.8 to 0.9) after one year.

**Conclusions:**

The mean level of posttraumatic stress symptoms in patients one year following ICU treatment was high and one of four were above case level Predictors of posttraumatic stress symptoms were mainly demographics and experiences during hospital stay whereas clinical injury related variables were insignificant. Pessimism was a predictor of posttraumatic stress, anxiety and depression symptoms. A subgroup of patients developed clinically significant distress symptoms during the follow-up period.

## Introduction

Survivors of intensive care unit (ICU) treatment may experience psychological distress for some time after discharge from the ICU [1-3]. The reported prevalence of anxiety ranges from 12% to 43% [4,5], 10% to 30% for depression [4-6] and 5% to 64% [3] for posttraumatic stress disorder (PTSD)-related symptoms. Symptoms present a short time after ICU stay may decline as time goes by, whereas symptoms present at long-term follow up may be persistent [7]. Long-term data of the course of psychological distress symptoms in ICU survivors are limited [8].

Earlier publications have studied trauma, surgical and medical ICU patients separately with differing times of assessment [2,3,9,10]. Trauma and surgical patients may differ from medical patients due to the likelihood that PTSD-related symptoms experienced by these patients could be related to the trauma itself and/or surgical intervention. In a previous publication, we found that experiences due to treatment in the ICU, such as pain, lack of control and inability to express needs, were predictors of psychological distress symptoms a short time after ICU discharge [11]. Personality may also influence the course of psychological symptoms after intensive care treatment. Patients with an optimistic personality trait differ from pessimists in coping with serious disease; they recover more rapidly, have less psychological distress and have better quality of life [11-13]. It is not known whether different factors predict psychological distress in ICU survivors at short-term versus long-term follow-up periods. In the present study we explore factors that may influence psychological distress symptoms at one-year post ICU discharge.

### Aims

The aims of this study were to explore: the level of posttraumatic stress symptoms, anxiety and depression during the first year post ICU discharge in a mixed ICU population; differences in posttraumatic stress, anxiety and depression in medical, surgical and trauma patients; and the association between these psychological distress symptoms at one year post ICU discharge and patients characteristics (demographics, personality trait, clinical variables) and experiences during intensive care treatment.

## Materials and methods

This prospective cohort study was designed to examine psychological outcomes of survivors of critical illness. The patients were enrolled from February 2005 to December 2006. Oslo University Hospital, Ullevaal, is an academic, tertiary-care centre with an 11-bed general ICU, a six-bed medical ICU and a coronary unit with three beds for mechanically ventilated coronary patients. During a patient's stay, one physician and one team of nurses are assigned to the patient. Physical restraint is not used. During mechanical ventilation (MV), the patients are treated with sedatives and analgesics. Patients aged 18 to 75 years who had stayed at least 24 hours in the ICU were included in the study. Patients with language difficulties, major psychiatric illness (i.e. psychosis), severe head injury or cognitive failure were excluded. Patients with other pre-existing mental illnesses were not excluded. The Regional Ethics Committee and the Data Inspectorate approved the study.

### Assessment of patient characteristics

Pretrauma variables were demographic variables (age, gender, social status, education status, employment status and care for children) and personality traits. Clinical variables were disease category (trauma, medical, surgical and head injury/disease), Simplified Acute Physiology Score (SAPS) II [14], Nine Equivalent of Nursing Manpower use score (NEMS) [15], MV, duration of MV and length of stay in the ICU (LOS ICU).

### Questionnaires at 4 to 6 weeks, 3 and 12 months after ICU stay

All patients signed written informed consent. For patients who remained at the hospital, written information, a consent letter and a questionnaire were sent by mail to the rehabilitation hospital or sent home to the patients about four weeks after ICU discharge. For those transferred to other hospital ICUs, the questionnaire was sent after about six weeks. We assumed that at this time they were able to read the information letter and decide whether they wanted to participate or not.

The patients were asked about memory of pain, distress from lack of control, and inability to express needs. Response options were rated on a five-point Likert-scale from 0 (not at all) to 4 (to a very high degree).

The ICU memory tool has been used in previous studies to measure various aspects of memory after intensive care and it has been primarily tested and validated on ICU patients in England and Italy [16-19]. It consists of items about memory on admission to hospital and memory for the ICU stay. Memories from ICU stay are divided into having: memories of feelings (being uncomfortable, feeling confused, feeling down, feeling anxious/frightened, panic, pain); delusional memories (feeling that people were trying to hurt them, hallucination, nightmares, dreams); and factual recall (family, alarms, voices, lights, faces, breathing tube, suctioning, darkness, clock, tube in the nose, ward round).

The revised Life Orientation Test (LOT) is a scale measuring a pessimistic versus optimistic personality trait [20]. Personality trait in this study is thus defined as a measure of dispositional optimism versus degree of pessimism reflecting generalized outcome expectancies. The dispositional perspective is based on the idea that people have relatively stable qualities [21]. Ten items compose the revised LOT; four of the items are filler items and are not used in the scoring. The six items scored are summed to compute an overall personality trait score, which can range from 0 to 24, where a high score indicates optimism.

The Impact of Event Scale (IES) has two subscales (seven items on intrusion and eight items on avoidance)[22]. Each item is scored from 0 to 5, so the total score can range from 0 to 75. Higher scores indicate more severe PTSD-related symptoms. A score above 20 indicates reactions of clinical importance and a score above 35 indicates severe symptoms with high a probability of a PTSD diagnosis [23]. The Hospital Anxiety and Depression scale (HADS) [24] questionnaire consists of 14 items, seven for anxiety and seven for depression. The HADS instrument was found to perform well in assessing the symptom severity and case level of anxiety disorders and depression in somatic patients and gives clinically meaningful results as a psychological screening tool [25,26]. Each item is scored from 0 to 3, so that the maximum score is 21 on each of the HADS subscales. Each patient may be allocated to one of three categories for anxiety and depression, based on individual final scores: 0 to 7 = non-case; 8 to 10 = borderline case; and 11 or more = definite case.

The pattern of distress symptoms across time following a traumatic event has been described as chronic/persistent symptoms, delayed onset of symptoms, recovery of symptoms or resilience (no symptoms of distress) [7,8]. To explore differences in psychological distress score across time, patients score at baseline and 12 months were used to categorize the patients as recovering (decreasing score; IES-score ≥ 20 at 4 to 6 weeks and <20 at 12 months), resilience (stable low score, <20, at both time points), persistent symptoms (stable high score, ≥ 20, at both time points) or delayed symptoms (increasing score; <20 at 4 to 6 weeks and ≥ 20 at 12 months). One hundred and seventy patients had an IES-total score at both baseline and 12 months. The score at three months is used to indicate the course of symptoms.

At the first assessment, four to six weeks after ICU discharge, further referred to as baseline, questionnaires about ICU memories, LOT, HADS and IES were included. During follow-up, LOT, HADS and IES were assessed at both 3 and 12 months. One missing item was accepted in each subscale of IES and HADS, and on the LOT score. The missing item was replaced with the mean of the other items for that patient. Although one missing item was accepted some patients did not get a sum score on these scales resulting in 180 patients with an IES-total score at 12 months and 192 patients with HADS score. In this paper we refer to the highest cut-off score (IES ≥ 35, HADS ≥ 11) concerning symptom levels that probably needs treatment (case level).

### Statistical methods

Statistical analyses were performed with the SPSS for Windows Version 15.0, Illinois, Chicago, USA. Continuous variables are presented as mean scores with 95% confidence interval (CI). The significance level was set at *P *< 0.05. Independent sample t-tests were used when comparing two groups on normally distributed variables. For categorical variables, Pearson's Chi-squared test was used. The Friedman Test was used for repeated measures analyses of variance. Correlations between pairs of continuous variables were calculated using Spearman's correlation coefficients. When the aim was to identify variables independently and significantly associated with IES (case level ≥ 35), HADS-Anxiety or HADS-Depression (both case level ≥ 11), logistic regression analysis was used. In these analyses we adjusted for age and gender. Variables that were significantly associated with the dependent variable in the univariate analyses (*P *< 0.2) were included in a multivariable logistic regression model, using forward Wald variable selection. All independent variables included correlated below 0.7.

## Results

**Figure 1:**
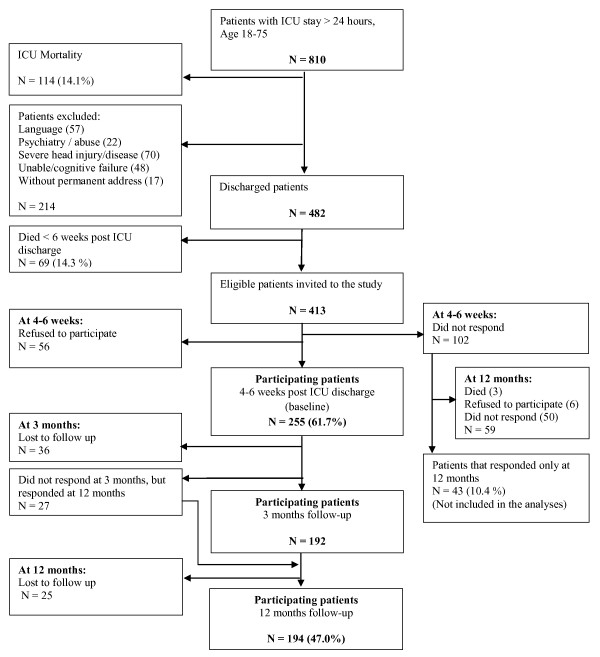
**Patient study recruitment diagram**. ICU, intensive care unit.

A total of 255 (61.7%) patients completed the first questionnaire and 194 of these completed the study at 12 months (Figure 1 and Table 1). Although 27 of the 194 patients did not respond to the three-month questionnaire, we have chosen to include these 27 patients in order not to lose information. We therefore used the 194 patients in the further analyses. Patients lost to follow up (total n = 61; 35 at 3 months and 26 at 12 months) were younger, had lower educational status and were more often unemployed before the ICU stay compared with those who completed the study at 12 months (n = 194), but they did not differ in clinical characteristics.

**Table 1:** Patient characteristics

Parameter	Value
Number of patients	255
Male gender, n (%)	160 (62.7)
Age, years mean (SD)	47.9 (15.7)
Men	45.7 (15.2)
Women	51.5 (15.7)
Marital status, n (%)	
- Married	105 (41.0)
- Single	97 (37.9)
- Living together	38 (15.2)
- Widow	10 (3.9)
- Other	5 (2.0)
Educational status, n (%)	
Primary school	41 (16.1)
Upper secondary education	142 (55.9)
College/university	71 (28.0)
Employment status, n (%)	
Working/student/retired	195 (76.5)
Unemployed/disabled	60 (23.5)
SAPS^1 ^score, mean (CI)	37.0 (35.3 to 38.7)
NEMS^2^, mean (CI)	29.6 (28.8 to 30.5)
LOS ICU^3^, mean days (CI)	12.0 (10.3 to 13.8)
MV^4 ^n (%)	216 (84.7)
Duration of MV, mean days (CI)	11.0 (9.3 to 12.7)
Disease category, n (%)	
Medical^5^	107 (42.0)
Surgical without trauma	62 (24.3)
Trauma^6^	86 (33.7)
Mild/moderate head injury/disease, n (%)	72 (28.2)
Transferred to local hospitals ICU, n (%)	132 (51.8)
Transferred while still on MV, n (%)	66 (25.9)
LOT^7^, mean (CI)	15.7 (15.1 to 16.3)

Mean with standard deviation (SD) or confidence intervals (CI), or n with percent (%).

^1^SAPS-2, Simplified Acute Physiology Score 2, measured during the first 24 hours of stay in the ICU.

^2^NEMS, Nine equivalents of nursing manpower use score presented as mean NEMS per day.

^3^LOS ICU, Length of stay in intensive care unit at Oslo University Hospital.

^4^MV, Mechanically ventilated.

^5^Medical: no surgical treatment during the ICU stay.

^6^Trauma: transport accident, fall accident, violence, sport/leisure time accidents/working accidents/other.

^7^LOT, Life Orientation Test.

A total of 112 (27%) patients either refused to participate or did not respond. These patients were significantly younger (42.4 years standard deviation (SD) 15.5 vs. 47.7 years SD 15.6, *P *= 0.003) and were more often transferred to local hospitals while still on MV, (49.1% vs. 26.6%, *P *= 0.001) than the patients that participated at four to six weeks (n = 255), but did not differ according to clinical variables.

In the present study, the 43 patients who participated only at 12 months lack baseline data and were not included in the regression analyses, in the analyses of the course of symptoms or in the analyses of prevalence. The results from these patients (n = 43) were only used for comparisons with the responders (n = 194). These patients were probably more seriously ill during the ICU stay because they had higher mean NEMS score (32.0, 95%CI = 30.4 to 33.7, vs. 29.6, 95% CI = 28.8 to 30.5; *P *= 0.04), were more often MV (97.7% vs. 84.7%, *P *= 0.02), had longer duration (days) of MV (16.2, 95% CI = 11.7 to 20.6, vs. 11.0, 95% CI = 9.3 to 12.7; *P *= 0.02) and were more often trauma patients (48.8% vs. 33.7%, *P *= 0.04) compared with the patients that participated at one month. No significant differences were found in age, gender, SAPS, LOS ICU, head injury/diseases or the proportion of patients that were transferred to other hospitals. The 43 patients did not differ significantly from the 194 patients at the one-year measurements of IES-total (21.9 vs. 22.5), HADS-Anxiety (6.8 vs. 5.8) or HADS-Depression (5.4 vs. 4.4) scores.

### The level of psychological distress

The mean score for IES-total one-year after ICU discharge (Table 2) was not significantly different between genders, but woman had higher scores than men (25.4 for women, 95% CI = 20.8 to 30.0, vs. 20.8 for men, 95% CI = 17.7 to 23.9; *P *= 0.086). Twenty-seven percent of the patients had scores at PTSD level at one year (IES-total ≥ 35; Table 2). No significant differences in psychological distress symptoms were seen between medical, surgical and trauma patients at one year, except that slightly more surgical patients had a HADS-Depression score of 11 or more compared with medical and trauma patients.

**Table 2:** Psychological distress measurements at one year

	All	Medical	Surgical	Trauma
IES^1 ^total, mean (CI)				
	22.5 (20.0 to 25.1)	22.8 (19.0 to 26.6)	22.3 (16.7 to 27.9)	22.4 (17.8 to 27.0)
IES-total				
≥ 20, %	50% (90/180)	51% (39/76)	46% (21/46)	52% (30/58)
≥ 35, %	27% (48/180)	25% (19/76)	33% (15/46)	24% (14/58)
HADS^2^				
Anxiety, mean (CI)	5.8 (5.1 to 6.5)	5.9 (4.9 to 6.9)	6.3 (4.8 to 7.8)	5.2 (3.9 to 6.5)
Depression, mean (CI)	4.7 (4.1 to 5.3)	4.4 (3.5 to 5.3)	5.6 (4.3 to 6.9)	4.3 (3.2 to 5.4)
HADS-Anxiety				
≥ 8, %	33% (63/192)	34% (28/82)	39% (20/51)	25% (15/59)
≥ 11, %	18% (35/192)	16% (13/82)	22% (11/51)	19% (11/59)
HADS-Depression				
≥ 8, %	27% (52/192)	23% (19/82)	37% (19/51)	24% (14/59)
≥ 11, %	12% (22/192)	9% (7/82)	22% * (11/51)	7% (4/59)

^1^IES, Impact of Event Scale.

^2^HADS, Hospital Anxiety and Depression scale.

* *P *< 0.05 between surgical and medical/trauma patients.

**Figure 2:**
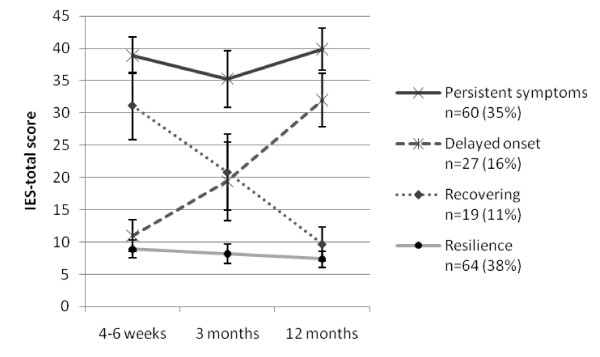
**Scores of posttraumatic stress symptoms during the first year**. Due to missing items, 170 patients had a score at baseline and 12 months. Eighteen of these did not respond at three months (six missing in each of the groups delayed onset and resilience, sixteen missing in the group with persisting symptoms). The score at three months is used to indicate the course of symptoms. IES, Impact of Event Scale.

During the first year following ICU discharge no differences in the IES-total, HADS-Anxiety and HADS-Depression mean scores across the three time points were found (Friedman, *P *= 0.388, *P *= 0.076, *P *= 0.446, respectively). Neither did we find any difference in the percentage of patients with symptoms above the lowest cut-off value, IES-total of 20 or more, between baseline (46%) and 12 months (51%; n = 170; Figure 2). At one year 16% (27 of 170) patients changed their IES-total score from IES-total less than 20 at four to six weeks to 20 or more at 12 months, further referred as delayed onset of symptoms. The mean IES score in this subgroup increased from 11 to 32 (Figure 2). The proportion of patients with delayed onset was not different in medical, surgical or trauma patients (Chi-Squared test = 0.565). Thirty-five percent of the patients had persistent symptoms during follow up, whereas 38% never showed any sign of posttraumatic stress symptoms.

Patients that were lost to follow up (n = 61) scored significantly higher on HADS-Anxiety at baseline compared with those who completed follow up (6.6 vs. 5.3, *P *= 0.041), but not significantly different on HADS-Depression (5.5 vs. 4.5, *P *= 0.116) or IES-total (25.0 vs. 21.8, *P *= 0.207). Patients that did not respond at 3 months (n = 27) had significantly higher IES-total mean score at 12 months compared with patients that answered at all three measure points (n = 167; 31.7 vs. 21.0, *P *= 0.004), but not significantly different anxiety (6.6 vs. 5.6) and depression (5.8 vs. 4.5) scores.

### Predictive factors for psychological distress symptoms at one year

In the univariate analyses, several variables were significantly associated with the IES-total of 35 or more at one year (Table 3). Adjusted for age and gender, low educational level, personality trait (pessimism), memory of pain and factual recall were independent predictors of posttraumatic stress symptoms. The subsequent multivariate model showed a good fit to the data, with a Hosmer-lemeshow statistic of 4.93 of 8 degrees of freedom (*P *= 0.77). Explained variance in the multivariate model by Cox/Snell and Nagelkerke R Square was 0.16 to 0.24. Stratified analyses by gender revealed no differences in predictive factors.

**Table 3:** Predictors of posttraumatic stress symptoms at one-year post ICU treatment

	**Univariate**^1^			**Multivariate**^2^		
		
	OR	CI	*P *value	OR	CI	*P *value
Age	1.00	0.98 to 1.02	0.811			
Gender	1.45	0.74 to 2.85	0.276			
Educational status^3^	0.33	0.14 to 0.76	0.009	0.38	0.15 to 0.95	0.038
Employment status^4^	2.55	1.17 to 5.52	0.018			
Personality trait^5^	0.92	0.86 to 0.99	0.019	0.91	0.84 to 0.99	0.029
Memory of pain	1.49	1.14 to 1.96	0.004	1.46	1.05 to 2.04	0.025
Lack of control	1.41	1.05 to 1.89	0.021			
Factual recall	5.50	1.86 to 16.29	0.002	6.61	1.41 to 30.97	0.017
Memory of feelings	1.77	0.90 to 3.48	0.098			
Delusional memories	1.88	0.96 to 3.66	0.064			
Cox & Snell R^2^/Nagelkerke R^2^				0.16/0.24		

Not all patients answered every question. Therefore, 159 of the 194 patients were included in the multivariate analyses. Age and gender were controlled for in the multivariate analyses.

^1^Univariate variables *P *< 0.20, age and gender are shown.

^2^Multivariable analysis, in *P *< 0.20, out *P *< 0.05.

^3^Educational status: low = 0, high = 1.

^4^Employment status before ICU stay: employed = 0, unemployed = 1.

^5^Personality trait: pessimism = low score; optimism = high score.

CI, confidence interval; OR, odds ratio.

To explore factors associated with delayed onset of posttraumatic stress symptoms multivariate regression analyses were performed. Twenty-seven patients were cases in this analysis (delayed onset; IES-total score <20 at 4 to 6 weeks and ≥ 20 at 12 months). Predictors for delayed onset of symptoms, adjusted for age and gender, were: unemployment (odds ratio (OR) = 3.1, 95% CI = 1.1 to 8.7, *P *= 0.035), LOS ICU (OR = 1.1, 95% CI = 1.0 to 1.1, *P *= 0.005), MV (OR = 0.3, 95% CI = 0.1 to 0.8, *P *= 0.014) and personality trait (optimism) (OR = 1.1, 95% CI = 1.0 to 1.3, *P *= 0.028; Nagelkerke R Square = 0.21).

Several variables were significantly associated with HADS-Anxiety in the univariate analyses at one year. Adjusted for age and gender, we found that unemployment (OR = 2.9, 95% CI = 1.2 to 7.1, *P *= 0.020), personality trait (optimism) (OR = 0.8, 95% CI = 0.8 to 0.9, *P *< 0.001) were independent predictors of anxiety symptoms (n = 187, Nagelkerke R^2 ^= 0.24). For HADS-Depression personality trait (optimism) (OR = 0.8, 95% CI = 0.7 to 0.9, *P *< 0.001) and surgery (OR = 4.0, 95% CI = 1.3 to 12.2, *P *= 0.013) were predictors (n = 187, Nagelkerke R^2 ^= 0.32).

In this study the LOT score did not differ during the three measure points, using paired sample t-test between baseline and 3 months (15.9 to 15.5, *P *= 0.153) and between 3 and 12 months (15.5 to 15.5, *P *= 0.832).

## Discussion

In the largest follow-up study to date in terms of the number of the ICU survivors, we found a high prevalence (27%) of patients above case level for posttraumatic stress (IES-total ≥ 35). PTSD risk during the first year following ICU discharge did not differ between medical, surgical and trauma patients. We also found that half of the patients had PTSD-related symptoms that might be of clinical significance (IES-total ≥ 20) one year after intensive care treatment. Furthermore, our results show that patients have different courses of symptoms post ICU-discharge; patients may have persistent symptoms, can recover, have delayed onset of symptoms or be resilience. This study is the first to show that a substantial proportion of ICU survivors (16%) may have delayed onset of posttraumatic stress symptoms of clinical significance, which strengthens the need for follow up of this population.

High levels of psychological distress found in our ICU patients support results of previous studies [2,3,27,28]. The mean level of psychological distress did not change significantly during the first year after trauma and this is in contrast to earlier reports [29]. Only two studies from general ICUs assessed PTSD-related symptoms in the same patients longitudinally. One study found no difference in anxiety, depression or posttraumatic stress symptoms between 3 and 9 months [30]. The other study found no difference in IES score between discharge and 6/12 months, but anxiety and depression scores were significantly reduced between hospital discharge and 6 months, but with no further reduction between 6 and 12 months [31].

Delayed PTSD was found to occur in 5 to 10% of trauma-exposed individuals and was associated with poorer social support [8,32,33]. However, only one of these studies was performed in ICU patients. One reason for a delayed onset of posttraumatic symptoms in ICU survivors may be due to the serious physical illness they must recover from and/or that the focus on physical recovery suppresses psychological symptoms. A rise in anxiety and depression symptoms over the first year after discharge could also be related to the initial hopefulness of recovery and then eventual realization of loss of function and/or potential and anxiety about the future. Our study supports the hypothesis that patients with persistent symptoms at three months would rarely spontaneously recover in the further course, and that patients that initially had no symptoms but showed a delayed response may remain symptomatic in the long term [7,33].

A substantial proportion of patients did not participate at all three measure points. In clinical follow-up studies, there are always some patients that do not respond at all time points. Accordingly, the data analyses carry risks of bias. By excluding subjects that do not respond at certain time points, some information is lost, and there is no gold standard for how to deal with this problem. We have therefore chosen to use all patients that responded at the first and last assessments. Among the 255 patients who were measured at baseline, 76% participated at 12 months, which is highly acceptable. We do not know the reasons for not participating. One reason may be suffering from psychological distress, confirmed by higher HADS-Anxiety score at baseline in those who were lost to follow up and higher IES-level at one year in those who did not respond at three months. However, patients who participated at one year only did not have significantly different IES scores from those with several assessments. Another reason for not participating may be that the patient was unable due to their physical impairment/limitations; however, we have no data to confirm such a possibility. The patients that participated at 12 months only were probably more seriously ill during the ICU stay and they might not have been able to answer at the first assessment. This show that studies initiated shortly after ICU treatment may risk losing those who are most severely injured. The results of this study show the importance of following up patients and assessing psychological distress until a stable recovery is achieved.

The large number of participants in this study made it possible to stratify patients into different disease categories. Previous studies of psychological distress in ICU survivors have focused on different disease categories separately (trauma, abdominal surgery, acute respiratory distress syndrome, sepsis, cardiac surgery or medical patients), while other studies have excluded surgical or trauma patients [1-3]. Different methodology and time of assessment between studies have made comparisons between disease categories difficult. Only one cross-sectional study that compared medical, surgical and trauma patients found no significant differences in the level of psychological distress between medical, surgical and trauma patients in accordance to our study [34]. Another study from a surgical ICU found a higher risk of developing PTSD in trauma than non-trauma patients [35].

Independent predictors of psychological distress in the long term differed at some points from the predictors found in the short term where; MV, pain and head injury together with patient demographics and experiences were significant [11]. The present study confirms that a personality trait of pessimism was a predictor posttraumatic stress, anxiety and depression symptoms in ICU patients also at long-term follow up. Predictors of posttraumatic stress symptoms at one year were demographics (low educational level), personality trait (pessimism) and experiences during stay (factual recall, memory of pain), whereas clinical injury variables were not significant. That severity of illness was not a predictor of distress at one year is supported by previous studies [2,27,31]. ICU patients may often be unaware of the degree of life-treat during treatment until the illness is largely resolved, but experiences during stay such as having factual recall and delusional memories were strong predictors in this study and are supported by others [27]. This study is the first to show that a memory of being distressed due to a lack of control during ICU treatment was a strong predictor for PTSD-related symptoms, anxiety and depression symptoms in ICU patients also at long-term follow up. Every effort during treatment to decrease the patient's distress due to lack of control should be a major goal.

### Limitations

The response rate in this study did not differ from comparable studies addressing the same topic in ICU survivors. Patients that refused to participate or did not respond may represent a source of bias. Nonparticipants were younger, but did not differ in other demographic or clinical variables compared with the participants. This may support the fact that there is a rather low probability of response bias in this study. Patients that were lost to follow up had more anxiety symptoms at baseline. Both psychological and physical impairments may be reasons for not participating in this study, but also patients that have fully recovered may also refuse to participate. The measurement of posttraumatic stress, anxiety and depression is performed with a self-report screening tool without the ability to diagnose any psychiatric disorder and there is a possibility to overestimate the magnitude of psychological distress. However, the aim of the study was to assess the level and course of symptoms during the first year after ICU discharge. A formal diagnosis of PTSD requires data on hyper arousal and the A-criterion, but the high the symptom levels found in this study are of clinical significance [36]. We found delayed onset of PTSD symptoms during follow up, but we did not ask the patients about new traumatic experiences post-ICU discharge. In any mailed self-administered questionnaires there is always a possibility that other persons may have influenced the participant when filling in their responses.

Another limitation of the study is the failure to measure prior psychological symptoms as this has been found to be a predictor in several studies [2,27,37]. In addition, no assessment of medication during ICU treatment, delirium during hospital stay or cognitive failure post ICU discharge was performed. The study was not designed as a multicentre study and as half of the patients were transferred to their local hospital ICU, assessment of medication, sedation level and delirium during ICU treatment became difficult. Delirium screening was performed in a pilot study but where we found a low degree of consciousness in most of our ICU patients due to medication we decided not to measure this in the present study. This may be considered a limitation as previous studies found that greater levels of sedation and delirium may cause PTSD-related symptoms.

## Conclusions

The mean level of posttraumatic stress symptoms in patients one year after ICU treatment was high and many patients, i.e., one of four, accordingly may need treatment. There was no difference in psychological stress between medical, surgical and trauma ICU patients. Predictors of posttraumatic stress symptoms were mainly demographics and experiences during stay whereas clinical variables were insignificant. The personality trait pessimism was a predictor of posttraumatic stress, anxiety and depression symptoms. A subgroup of patients developed clinically significant posttraumatic stress symptoms during the study period. Follow up of the psychological symptoms of ICU survivors seems important.

## Key messages

• One in four ICU survivors experience posttraumatic stress symptoms one year after ICU discharge.

• No differences in psychological distress between medical, surgical and trauma patients were seen.

• Pessimism was a predictor of posttraumatic stress, anxiety and depression symptoms.

• A subgroup of ICU survivors develops clinically significant posttraumatic stress symptoms during follow up.

## Abbreviations

CI: confidence interval; HADS: Hospital Anxiety and Depression Scale; ICU: intensive care unit; IES: Impact of Event Scale; LOT: Life Orientation Test; LOS: length of stay; MV: mechanical ventilation; NEMS: Nine Equivalents of Nursing Manpower Use score; OR: odds ratio; PTSD: posttraumatic stress disorder; SAPS: Simplified Acute Physiology Score II; SD: standard deviation.

## Competing interests

The authors declare that they have no competing interests.

## Authors' contributions

The authors HM, OS and ØE made substantial contributions to the conception and design of the study. HM, SK and KT completed the data collection. HM performed the study and the statistical analysis. All authors read and approved the final manuscript.
